# Mental health emergency presentations across the Barwon South West region in Victoria, Australia: An epidemiological investigation

**DOI:** 10.1111/1742-6723.14223

**Published:** 2023-04-24

**Authors:** Bianca E Kavanagh, Kara L Holloway‐Kew, Timothy Baker, Mohammedreza Mohebbi, Julie A Pasco, Kayla B Corney, Mark A Kotowicz, Shae E Quirk, Lana J Williams

**Affiliations:** ^1^ Deakin University Institute for Mental and Physical Health and Clinical Translation, School of Medicine, Barwon Health Geelong Victoria Australia; ^2^ Deakin Rural Health, Deakin University Warrnambool Victoria Australia; ^3^ Centre for Rural Emergency Medicine, Deakin University Warrnambool Victoria Australia; ^4^ Biostatistics Unit, Faculty of Health Deakin University Geelong Victoria Australia; ^5^ University Hospital Geelong, Barwon Health Geelong Victoria Australia; ^6^ Department of Medicine, Western Health The University of Melbourne Melbourne Victoria Australia; ^7^ Department of Epidemiology and Preventive Medicine Monash University Melbourne Victoria Australia

**Keywords:** Australia, emergency department, emergency presentations, mental health, rural, Victoria

## Abstract

**Objective:**

To examine mental health emergency presentations across the Barwon South West, Victoria, Australia – an area comprising a range of urban and rural localities.

**Methods:**

This is a retrospective synthesis of mental health emergency presentations across the Barwon South West (1 February 2017–31 December 2019). De‐identified data were obtained from individuals who presented to EDs and urgent care centres (UCCs) within the study region, who had a principal diagnosis of a *Mental and Behavioural Disorder* (codes F00‐F99). Data were sourced from the Victorian Emergency Minimum Dataset and Rural Acute Hospital Database Register (RAHDaR). Age‐standardised incident rates for mental health emergency presentations were calculated for the whole sample and for local government areas. Data on usual accommodation, arrival transport mode, referral source, patient disposition and length of ED/UCC stay were also obtained.

**Results:**

We identified 11 613 mental health emergency presentations, with neurotic, stress‐related and somatoform disorders (*n* = 3139, 27.0%) and mental and behavioural disorders due to psychoactive substance use (*n* = 3487, 30.0%) being the most frequent types of presentations recorded. The highest age‐standardised incidence rates (mental health diagnosis per 1000 population/year) were in Glenelg (13.95), whereas Queenscliffe had the lowest incident rates (3.76). Most presentations (*n* = 3851, 33.2%) tended to occur for individuals aged between 15 and 29 years.

**Conclusions:**

Neurotic, stress‐related and somatoform disorders and mental and behavioural disorders due to psychoactive substance use were the most frequent types of presentations recorded across the sample. RAHDaR represented a small but meaningful contribution to the data.

## Graphical abstract

The present study aimed to complete a retrospective synthesis of mental health emergency presentations across the Barwon South West (1 February 2017–31 December 2019), using data from the Victorian Emergency Minimum Dataset and Rural Acute Hospital Database Register. We identified 11 613 mental health emergency presentations. Neurotic, stress‐related and somatoform disorders and mental and behavioural disorders because of psychoactive substance use were the most frequent types of presentations recorded.

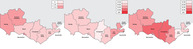

**Key findings**

- Across the Barwon South West region, neurotic, stress‐related and somatoform disorders and mental and behavioural disorders due to psychoactive substance use were the most common types of mental health emergency presentations recorded.
- The highest proportion of presentations tended to occur for adolescents and young adults.
- Non‐mandated rurally‐collected data adds an important contribution to the overall picture of mental health emergency presentations.

## Introduction

EDs are a critical element of mental health service provision, representing the nexus between community‐based and inpatient care, and are a central entry point to access healthcare for individuals experiencing mental health crises.^1^ This may be particularly evident in areas where the demand for mental health services is outweighed by their capacity to provide care,^2^ such as in rural localities. This is reflected in the rapid and consistent increase in mental health presentations in rural EDs (+65.2%), compared to principal referral (i.e. large hospitals which provide a comprehensive range of highly specialised services) (+47.6%), and major metropolitan (+43.2%) facilities over a 10‐year period.^3^ Other research, however, has demonstrated a similar number of mental health emergency presentations across metropolitan and rural/regional areas, yet patients from rural/regional areas experience longer waiting times for inpatient admission.^4^

In Victoria (Australia's second most populous state), EDs are staffed 24 h a day, 7 days per week by medical staff. EDs receive activity‐based funding, which requires the reporting of patient‐level data to the Victorian Emergency Minimum Dataset (VEMD).^5^ Smaller rural hospital‐based emergency care services – called urgent care centres (UCCs) in Victoria – have scope to manage emergencies, including performing stabilisation, resuscitation and preparing patient transfers to hospitals with capacity to provide a higher level of care.^5^ However, unlike EDs, UCCs receive block funding grants and are therefore not mandated to report to the VEMD.^5^ Consequently, this may result in inadequate assessment of rural healthcare needs.^6^ To meet this deficit, the Rural Acute Hospital Database Register (RAHDaR) was established and is designed to shadow the VEMD by reporting de‐identified episode‐level data gathered by 10 UCCs located in western Victoria.

To date, there has been limited epidemiological data of inconsistent quality that have specifically examined mental health emergency presentations in Australia.^7^ Simultaneously, a data deficit in rural health has been identified.^8^ Previous research has examined physical health emergency presentations from government‐collected and rural databases in south west Victoria^8^, ^9^, ^10^, ^11^ – a district within the larger western Victoria legislative region. The current study therefore aims to build on the previous work, with a specific focus on mental health emergency presentations among individuals with a principal mental health diagnosis, across the Barwon South West region of Victoria, Australia, using data generated from both the VEMD and RAHDaR.

## Methods

The current research is a sub‐study of the Ageing, Chronic Disease and Injury (ACDI) study, which seeks to map chronic disease and injury across western Victoria, Australia.^12^ The present study is focussed on a sub‐region of western Victoria, known as Barwon South West.

### Study setting

This retrospective synthesis study utilised de‐identified mental health emergency presentation data from children, adolescents, and adults between 1 February 2017 and 31 December 2019 (2.92 years). Data were collected from EDs and UCCs located within the Barwon South West region of Victoria, Australia. This area is encompassed by 21 of the 79 Victorian local government areas (LGAs), which represent distinct geographical regions, covering ~29 000 km^2^. Barwon South West comprises of a range of urban and rural areas, and has an estimated population of 428 551 individuals.^13^

### Data collection

Data on de‐identified episode‐level mental health emergency presentations were obtained from the VEMD and RAHDaR. The VEMD includes the following EDs situated within larger hospitals in Barwon South West, Victoria: Hamilton Base Hospital, University Hospital Geelong and Warrnambool Base Hospital. These hospitals pertain to the Southern Grampians, Greater Geelong and Warrnambool LGAs, which represent small and medium rural towns, metropolitan, and large rural towns, respectively.^14^ In addition, the LGA of Queenscliffe (a large rural town) is most closely located to University Hospital Geelong (32 km distance), where patients are likely to travel to for emergency care. RAHDaR provided data for UCCs at the following smaller hospitals: Colac Area Health, Camperdown Hospital, Lorne Community Health, Moyne Health Services, Otway Health and Community Services, Portland District Health, Terang Health Service and Timboon District Health, and comprise the Colac‐Otway (medium and large rural towns), Corangamite (small rural towns), Surf Coast (regional centre, small rural towns and large rural towns), Moyne (small and large rural towns) and Glenelg (small and medium rural towns) LGAs.^14^ At present, RAHDaR does not include any data from UCCs located at Casterton or Heywood, both located within the Glenelg LGA; consequently, these presentations are omitted from the current study. Aggregate data from both sources were combined by the research team.

Paediatric, adolescent and adult emergency presentation episodes were extracted for all patients who had a principal diagnosis of a *Chapter 5 Mental and Behavioural Disorder* according to the International Classification of Disease‐10 (ICD‐10) (codes F00‐F99). Information on usual accommodation, arrival transport mode, referral source, patient disposition and length of stay (LoS) in ED/UCC were also obtained. Data were based on the VEMD version 25 for 2020–2021.

### Statistical analyses

Direct age‐standardised incidence rates for mental health ED presentations were calculated for each LGA using population data from the Australian Bureau of Statistics (ABS) 2016 Census Community Profile Series.^13^ Data are reported as incidence rates (mental health diagnosis per 1000 population/year). Poisson regression analyses were performed to compare the incidence of mental health emergency presentations across LGAs. Data pertaining to usual accommodation, arrival transport mode, referral source and patient disposition were explored for the whole sample and the most frequent characteristics identified are reported. Characteristics of the sample were compared using chi‐squared and Kruskall Wallis tests for categorical variables, and analysis of variance for continuous variables. Data were analysed using StataSE 16.^15^

### Ethics approval

This research was carried out in accordance with the latest version of the Declaration of Helsinki and approved by the Barwon Health Human Research Ethics Committee (HREC) (reference no. 15/11), South West Healthcare HREC (2021 17) and Deakin University HREC (2015‐117).

## Results

There were 10 343 mental health emergency presentations obtained from the VEMD dataset. Of those, 37 presentations were excluded as it was not possible to decipher with which LGA they were associated. Four further presentations were excluded as they pertained to individuals who identified their sex as ‘other’ and population statistics were only available for those who identified as males or females. For the UCCs included in RAHDaR, 1321 presentations were obtained and 10 were removed due to participants being located outside the study region. Consequently, 11 613 presentations between 1 February 2017 and 31 December 2019 were included in the final analysis.

### Characteristics of the presentations

**Figure 1:**
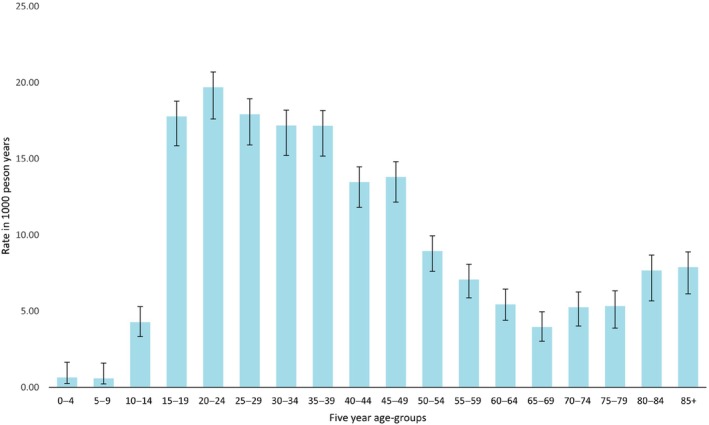
Age‐standardised incident rates (direct to the 2016 Australian population, per 1000 population/year) for mental health emergency presentations in Barwon South West.

Of the 11 613 presentations, 5807 (50.0%) were for females and 5806 (50.0%) were for males. Across the study region, Poisson regression analyses revealed that the age‐standardised incidence rates were 10.1 for females, 10.7 for males and 10.4 per 1000 population/year for females and males combined (Table 1). The age‐standardised incident rates revealed that most presentations tended to occur in the 15–19, 20–24 and 25–29‐year age groups (Fig. 1).

**TABLE 1:** Age‐standardised mental health emergency presentations between 2017 and 2019 according to local government area and sex

LGA	Female	Male	Total
Colac‐Otway†	10.9	8.3	9.5
Corangamite†	9.8	9.7	9.7
Glenelg†	13.0	15.3	14.0
Greater Geelong‡	10.4	11.3	10.8
Moyne†	7.4	7.9	7.6
Queenscliffe†	2.0	5.8	3.8
Southern Grampians‡	11.7	10.6	11.1
Surf Coast†	5.5	6.8	6.1
Warrnambool‡	12.0	12.7	12.3
Total	10.1	10.7	10.4

Data presented as 1000 persons/year.

† Data derived from Rural Acute Hospital Database Register.

‡ Data derived from Victorian Emergency Minimum Dataset.

Neurotic, stress‐related and somatoform disorders (*n* = 3139, 27.0%) and mental and behavioural disorders due to psychoactive substance use (*n* = 3487, 30.0%) were the most frequent types of presentations recorded across the sample. Significant differences in the types of mental disorder presentations were identified between the sexes (*P* < 0.001) (Table 2). Males tended to have more emergency presentations for mental and behavioural disorders due to psychoactive substance use (*n* = 2073, 35.7%) and schizophrenia, schizotypal and delusional disorders (*n* = 932, 16.1%). Females had more presentations for organic, including symptomatic, mental disorders (*n* = 397, 6.8%), of which females had a higher proportion of delirium (females *n* = 337, males *n* = 237), but not dementia (females *n* = 60, males *n* = 78), compared to males. In addition, females had more presentations for neurotic, stress‐related, and somatoform disorders (*n* = 1865, 32.1%), disorders of adult personality and behaviour (*n* = 258, 4.4%) and intentional self‐harm (*n* = 834, 14.4%).

**TABLE 2:** Mental health emergency presentations according to ICD‐10‐AM principal diagnosis and intentional self‐harm

Block ICD‐10‐AM principal diagnoses		Females (*n* = 3075)	Males (*n* = 2864)	Total (*n* = 5939)
		*n* (%)	*n* (%)	*n* (%)
F00‐F09	Organic, including symptomatic, mental disorders	397 (6.8)	315 (5.4)	712 (6.1)
F10‐F19	Mental and behavioural disorders due to psychoactive substance use	1414 (24.4)	2073 (35.7)	3487 (30.0)
F20‐F29	Schizophrenia, schizotypal and delusional disorders	511 (8.8)	932 (16.1)	1443 (12.4)
F30‐F39	Mood (affective) disorders	850 (14.6)	752 (13.0)	1602 (13.8)
F40‐F48	Neurotic, stress‐related and somatoform disorders	1865 (32.1)	1274 (21.9)	3139 (27.0)
F50‐F59	Behavioural syndromes associated with psychological disturbances and physical factors	99 (1.7)	†	103 (0.9)
F60‐F69	Disorders of adult personality and behaviour	258 (4.4)	67 (1.2)	325 (2.8)
F70‐F79	Mental retardation	14 (0.2)	11 (0.2)	25 (0.2)
F80‐F89	Disorders of psychological development	10 (0.2)	9 (0.2)	19 (0.2)
F90‐F98	Behavioural and emotional disorders with onset usually occurring in childhood and adolescence	174 (3.0)	151 (2.6)	325 (2.8)
F99	Mental disorder not otherwise specified	215 (3.7)	218 (3.8)	433 (3.7)
‐	Intentional self‐harm‡	834 (14.4)	641 (11.0)	1475 (12.7)

† Data not reported due to <5 cases.

‡ Includes suicidal and non‐suicidal intent.

### Local government areas

**Figure 2:**
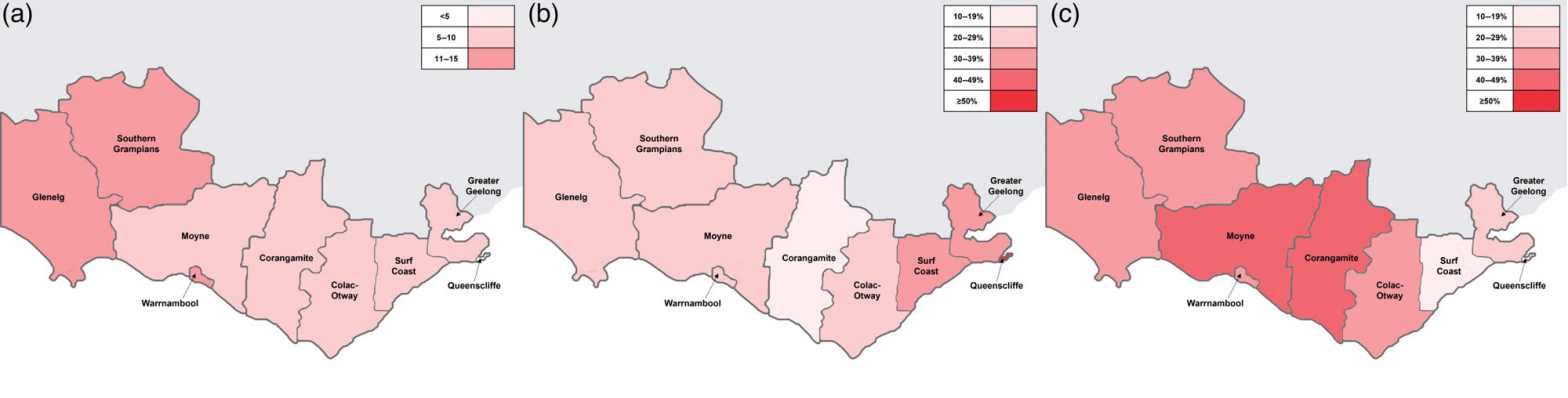
Heat map of (a) the age‐standardised incident rates (per 1000 population/year) for mental health emergency presentations; (b) proportion of neurotic, stress and somatoform disorders (% [of total for local government area]); and (c) proportion of mental and behavioural disorders due to psychoactive substance use (% [of total for local government area]) for each local government area across Barwon South West, Victoria. The denominator for each proportion is the number of presentations for the corresponding local government area.

The age‐standardised incident rates of mental health emergency presentations were varied across the LGAs – these data are reported in Table 2 and Figure 2. The highest incidence rates were found in Glenelg (13.95) per 1000 population/year, followed by Warrnambool (12.33). Queenscliffe had the lowest incident rates (3.76). The age‐standardised incidence rates were higher for females in Colac‐Otway (10.87), compared with males (8.25), whereas Greater Geelong (11.32) and Queenscliffe (5.75) had higher rates for males compared with females (10.35; 1.95, respectively). In terms of presentations according to age, the highest incident rates were found in Glenelg in the 25–29‐year age‐group (40.68) (Table S1).

The highest proportion of presentations across the LGAs tended to occur for either neurotic, stress‐related and somatoform disorders or mental and behavioural disorders due to psychoactive substance use. Corangamite had the highest proportion of emergency presentations for neurotic, stress‐related, and somatoform disorders (*n* = 200, 48.7%) and the lowest proportion of mental and behavioural disorders due to psychoactive substance use (*n* = 81, 19.7%). Surf Coast had the lowest proportion of emergency presentations for neurotic, stress‐related and somatoform disorders (*n* = 89, 19.7%), whereas Queenscliffe had the highest proportion of mental and behavioural disorders due to psychoactive substance use (*n* = 14, 58.3%). Heatmaps displaying the proportion of presentations across the LGAs are displayed in Figure 2. Block diagnoses by each LGA can be found in Table S2.

Among the LGAs, differences were identified in patient's usual accommodation, arrival transport mode, referral source and patient disposition (all *P* < 0.001) and LoS (*F*(8, 11 612) = 26.73, *P* < 0.001). Most patients lived in a private residence; arrived by road ambulance or other, including private car; were referred by self, family, or friends; and did not have an applicable disposition post‐ED presentation. Median LoS in ED/UCC was highest for patients from Queenscliffe (240 min, range 169–372 min) and shortest for patients from Corangamite (102 min, range 52–245 min) (Table 3).

**TABLE 3:** Emergency presentation characteristics of the sample according to LGA

LGA	Usual accommodation				Arrival transport mode				Referral source				Patient disposition				Length of stay#
	Private residence	Unknown	Aged care residential facility	Other	Road ambulance	Other, including private car	Police vehicle	All other	Self/family/friends	Correctional officer/other police	Prison/person in custodial care	Other	Not applicable	Local medical officer	Mental health community service	Other	
Colac‐Otway†	508 (92.7)	19 (3.5)	7 (1.3)	14 (2.6)	190 (34.7)	327 (59.7)	26 (4.7)	5 (0.9)	443 (80.8)	29 (5.3)	8 (1.5)	68 (12.4)	93 (17.0)	153 (27.9)	102 (18.6)	200 (36.5)	168 (89–319)
Corangamite†	389 (94.7)	9 (2.2)	§	9 (2.2)	142 (34.6)	247 (60.2)	18 (4.4)	§	325 (79.1)	16 (3.9)	5 (1.2)	65 (15.8)	83 (20.3)	143 (35.0)	50 (12.2)	133 (32.5)	102 (52–245)
Glenelg†	660 (95.7)	22 (3.2)	§	5 (0.7)	277 (40.1)	375 (54.4)	32 (4.6)	6 (0.9)	553 (80.1)	33 (4.5)	7 (1.0)	97 (14.1)	91 (13.2)	269 (39.0)	118 (17.1)	212 (30.7)	153 (87–270)
Greater Geelong‡	6468 (86.9)	401 (5.4)	181 (2.4)	396 (5.3)	3939 (52.9)	3059 (41.1)	403 (5.4)	45 (0.6)	5918 (79.5)	350 (4.7)	389 (5.2)	789 (10.6)	2887 (38.8)	1578 (21.2)	1663 (22.3)	1318 (17.7)	211 (130–350)
Moyne†	325 (95.3)	11 (3.2)	§	§	103 (30.2)	219 (64.2)	15 (4.4)	§	269 (78.9)	7 (2.1)	16 (4.7)	49 (14.4)	97 (28.5)	92 (27.0)	52 (15.3)	100 (29.3)	164 (101–273)
Queenscliffe†	24 (100.0)	§	§	§	14 (58.3)	9 (37.5)	§	§	19 (79.2)	§	§	§	14 (58.3)	§	§	§	240 (169–372)
Southern Grampians‡	465 (96.1)	§	5 (1.0)	11 (2.3)	163 (33.7)	281 (58.1)	34 (7.0)	6 (1.2)	395 (81.6)	12 (2.5)	15 (3.1)	62 (12.8)	137 (28.3)	144 (29.8)	84 (17.4)	119 (24.6)	150 (85–239)
Surf Coast†	420 (92.7)	15 (3.3)	11 (2.4)	7 (1.6)	239 (52.8)	198 (43.7)	15 (3.3)	§	369 (81.5)	18 (4.0)	13 (2.9)	53 (11.7)	176 (38.9)	116 (25.6)	82 (18.1)	79 (17.4)	218 (137–363)
Warrnambool‡	1160 (95.4)	31 (2.6)	12 (1.0)	13 (1.1)	441 (36.3)	698 (57.4)	71 (5.8)	6 (0.5)	956 (78.6)	49 (4.0)	46 (3.8)	165 (13.6)	344 (28.3)	335 (27.6)	250 (20.6)	287 (23.6)	169 (99–290)
Total¶	10 419 (89.7)	511 (4.4)	225 (1.9)	458 (3.9)	5508 (47.4)	5413 (46.6)	615 (5.3)	76 (0.7)	9247 (79.6)	516 (4.4)	499 (4.3)	1351 (11.6)	3922 (33.8)	2833 (24.4)	2405 (20.7)	2451 (21.1)	194 (115–327)

Values reported as *n* (% [of total for local government area]) or median (range).

† Data derived from Rural Acute Hospital Database Register.

‡ Data derived from Victorian Emergency Minimum Dataset.

§ Data not reported due to <5 cases.

¶ Total includes all cases regardless of data not reported.

# Length of stay in ED/UCC, rounded to the nearest minute.

## Discussion

Using data from the VEMD and RAHDaR, the present study investigated the mental health emergency presentations that occurred in the Barwon South West region of Victoria, Australia during a 2.92‐year period. A total of 11 613 mental health emergency presentations were identified (age‐standardised incidence rate 10.4). Of this, 1311 (11.3%) were obtained from hospitals reporting non‐mandated data to RAHDaR – representing a small, but meaningful contribution to the data.

Most mental health emergency presentations tended to occur for individuals aged 15–29 years. These data echo the pattern at a national level, with the highest rates of mental health emergency presentations occurring among individuals aged between 18 and 24 years.^16^ This coincides with reports of doubled rates of high or very high psychological distress among Australians aged 16–24 years, compared to those aged 65–85 years.^17^ Alarcon Manchego *et al*.,^18^ in a study of four Victorian EDs, identified an increase in the acuity of presentations seen in patients aged ≤25 years between 2004 and 2013. The onset of most mental health conditions tend to peak between ages 14.5 and 18 years^19^ – potentially increasing mental health emergency presentations among this age cohort. Further, although national services (i.e. Headspace) have been implemented to target the mental health of younger Australians, specific and targeted services should also be a priority at the local scale. This would help to address the increased mental distress and consequential emergency presentations among this group. These data reflect calls from global studies, highlighting the necessity for research to prioritise efforts in early mental health interventions.^19^, ^20^, ^21^

The most common reasons for mental health emergency presentations tended to be for neurotic, stress‐related and somatoform disorders or mental and behavioural disorders due to psychoactive substance use. These data are akin to presentations seen across Australia, with previous data demonstrating that over half of all mental health emergency presentations occurring for these disorders.^22^ Tran *et al*.^23^ observed a two‐fold increase in ED presentations for mental and behavioural disorders due to psychoactive substance use between 2004–2005 and 2016–2017 in Australia. In addition, the most common diagnostic group reported in Australia was neurotic, stress‐related, and somatoform disorders. These data suggest that particular attention needs to be given to preventing and treating such disorders before patients present to the EDs in crisis. Initiatives at the local level could steer targeted interventions to address specific needs within communities.

It is noteworthy that males had slightly higher age‐standardised incidence rates for mental health emergency presentations than females. This is despite females experiencing a higher level of psychological distress and being more likely to help‐seek for their mental health than males in Australia.^17^, ^24^ Recent Australian data reported that during 2017–2018, the rate of mental health‐related ED presentations were greater for males than females (i.e. 121.7 compared to 110.0 per 10 000 population, respectively)^16^ – similar data were also reported during the 2019–2020 period.^22^ In recent years, there has been concerted efforts to raise awareness for men's mental health in the community. In some cases, a specialised focus on men's mental health has been included or is a core aim of initiatives. This consists of global and local fundraising and awareness efforts (i.e. Movember) to recognise men's health issues. Such efforts may play a part in mental health help‐seeking via EDs/UCCs that has occurred for males in the current study.

Geographically, the age‐standardised incidence rates for mental health emergency presentations appeared to be spread across the district, with lower incidence rates appearing to occur in Queenscliffe and higher incidence rates occurring in Glenelg, with ~4 versus ~14 individuals per 1000 per population/year presenting to an ED/UCC for mental health reasons, respectively. The LGA of Glenelg has a population of 19 621 individuals^13^ and is encompassed by a medium rural town, surrounded by small rural towns. Collectively, small rural towns cover geographically vast areas, and are defined as areas that are more than a 10 km drive from a town that is populated by 5000 to 15 000 residents^14^ – these areas comprise the majority of the study region. Versace *et al*.^25^ acknowledged that the spatial distribution of small rural towns is similar to those of remote and very remote communities, but that these areas do not have healthcare services that are often situated in large and medium rural towns. Thus, individuals with acute mental health concerns may travel to large rural towns, such as Portland (the major town in Glenelg), to access more comprehensive emergency mental healthcare that may not be provided at UCCs. Further, geographical and social isolation, environmental and social challenges, reluctance to seek help,^26^, ^27^ and socioeconomic disadvantage^25^ may influence the incidence of mental health emergency presentations in rural areas.

With the exception of the Greater Geelong LGA, most patients were referred to local medical officers upon ED/UCC departure (where a referral was applicable), suggesting that much of the mental healthcare in more rural areas may be provided by general practitioners. Patients from Greater Geelong – the most urban LGA – however, were referred to local medical officers and community mental health services in almost equal proportions, suggesting community mental health services are more readily accessible in urban areas. These data reflect the disparity of the mental health workforce in urban compared to other remoteness areas.^28^ Moreover, the Greater Geelong LGA is additionally serviced by a specialist private mental health inpatient service, whereas other areas within the Barwon South West region are largely dependent on place‐based non‐government organisations and services provided hospitals and UCCs.

The present study has a number of strengths. We utilised data generated from both the VEMD and RAHDaR, rather than data extracted from medical records. The use of RAHDaR allowed the inclusion of emergency presentations that would have otherwise been omitted from our analyses. We investigated a vast geographical area, which represented a range of urban and rural localities. We also calculated age‐standardised emergency presentation rates for each LGA. Several limitations to the study must also be mentioned. We obtained aggregate data, which precludes specific interpretations at an individual level. We completed analyses using block ICD‐10 codes, and a nuanced interpretation of the specific disorders within these codes is not reported. There may be inaccuracies in the reporting of VEMD data (e.g. mental health concerns reported as physical health issues), as diagnostic codes are usually imputed by clinicians in busy environments with limited training in diagnostic coding and data entry.^29^ RAHDaR does not currently capture emergency presentations in two UCCs within the Glenelg LGA, and it is likely that presentations have been under‐reported in this area. Emergency presentations were also only obtained from those with a principal mental health diagnosis, and presentations where a mental health diagnosis was not assigned but may have been relevant are not included. Several LGAs within the study sample comprise a mix of rural and urban categories, and for this reason, incidence rates across different remoteness categories were unable to be directly compared. Last, the data from the present study are specific to Barwon South West, Victoria and may not be generalisable to the wider population – although many observations were consistent with nationally reported data.^16^, ^22^

## Conclusion

The current study demonstrated that the majority of mental health emergency presentations occur for neurotic, stress‐related and somatoform disorders or mental and behavioural disorders due to psychoactive substance use. The emergency presentations were dominated by adolescents and young adults, coinciding with the onset of the majority of mental health conditions.^19^ In general, these observations support the wider literature on mental health emergency presentations across Australia.

The present findings add to the scant epidemiological data on rural mental health emergency presentations in Australia and may serve to decrease the data deficit in this area. Given that the needs of health services in the community are identified through the outcomes of government‐mandated reporting mechanisms – such as the VEMD – it is possible that accurate data on the needs of rurally‐dwelling individuals are not readily known by the government. The use of rurally‐collected healthcare data can supplement these data and provide a more comprehensive analysis of the healthcare needs among local communities, having a direct effect on the health outcomes of the rural population. This may be particularly important in the mental health sector. Many mental health emergency presentations pertain to individuals who have unmet social and addiction needs;^30^ thus, having a complete picture of where local services need to target early intervention efforts is warranted.

## Supporting information


**Table S1.** Age‐standardised incident rates according to LGA and age‐group. Data presented as 1000 persons/year.

**Table S2.** Types of mental health presentations according to local government area, based on ICD‐10‐AM principal diagnosis and intentional self‐harm. Data are presented as number (% [of total for local government area]) of types of mental health presentations.

## Data Availability

The data that support the findings of this study are available from the Victorian Minimum Emergency Dataset and Rural Acute Hospital Database Register. Restrictions apply to the availability of these data, which were used under license for this study. Data are available from the authors with the permission of the Victorian Minimum Emergency Dataset and Rural Acute Hospital Database Register.
