# Supplementary material for: Mental health emergency presentations across the Barwon South West region in Victoria, Australia: An epidemiological investigation
Source: Emerg Med Australas. 2023 Apr 24;35(5):777–85. doi: 10.1111/1742-6723.14223 (PMC10947229; doi:10.1111/1742-6723.14223)
Supplement: Supplementary file 1 — Table S1. Age‐standardised incident rates according to LGA and age‐group. Data presented as 1000 persons/year. [file EMM-35-777-s001.docx]

**Supplemental Table 1.**

Age-standardised incident rates according to LGA and age-group. Data presented as 1,000 persons/year.

|  | **0-4** | **5-9** | **10-14** | **15-19** | **20-24** | **25-29** | **30-34** | **35-39** | **40-44** |
| --- | --- | --- | --- | --- | --- | --- | --- | --- | --- |
| Colac-Otway† | 1.1  (0.0, 2.3) | 1.3  (0.2, 2.5) | 2.1  (0.7, 3.6) | 15.1  (11.0, 19.1) | 19.3  (14.5, 24.1) | 19.3  (14.6, 24.0) | 15.7  (11.4, 20.0) | 13.7  (9.6, 17.7) | 10.9  (7.5, 14.3) |
| Corangamite† | 0.4  (-0.4, 1.1) | 1.3  (0.0, 2.5) | 9.0  (5.6, 12.4) | 9.8  (6.3, 13.3) | 11.6  (7.2, 16.1) | 17.8  (12.0, 23.5) | 17.8  (12.0, 23.7) | 17.5  (12.2, 22.9) | 13.0  (9.6, 18.7) |
| Glenelg† | 0.3  (-0.3, 1.0) | 1.0  (-0.1, 2.1) | 4.0  (1.9, 6.1) | 23.6  (18.5, 28.6) | 29.0  (22.6, 35.3) | 40.7  (32.4, 49.0) | 20.0  (14.7, 25.4) | 28.0  (21.8, 34.2) | 12.7  (9.0, 16.3) |
| Greater Geelong‡ | 0.5  (0.3, 0.7) | 0.5  (0.3, 0.7) | 4.7  (4.0, 5.4) | 18.3  (17.0, 19.5) | 18.9  (17.7, 20.2) | 16.8  (15.6, 18.0) | 18.6  (17.3, 19.8) | 18.3  (17.0, 19.6) | 15.0  (13.9, 16.1) |
| Moyne† | 1.0  (-0.1, 2.2) | 0.9  (-0.1, 1.8) | 4.1  (2.0, 6.2) | 14.7  (10.3, 19.1) | 13.9  (8.9, 18.8) | 13.9  (9.0, 18.8) | 10.0  (6.0, 14.0) | 12.5  (8.2, 16.7) | 5.8  (3.2, 8.4) |
| Queenscliffe† | 0.00  (0.0, 0.0) | 0.0  (0.0, 0.0) | 0.0  (0.0, 0.0) | 0.0  (0.0, 0.0) | 0.0  (0.0, 0.0) | 0.0  (0.0, 0.0) | 0.0  (0.0, 0.0) | 0.0  (0.0, 0.0) | 0.0  (0.0, 0.0) |
| Southern Grampians‡ | 2.8  (0.7, 4.8) | 0.7  (-0.3, 1.7) | 4.8  (2.3, 7.2) | 23.7  (18.2, 29.3) | 22.5  (16.3, 28.7) | 18.3  (12.5, 24.1) | 11.5  (7.2, 15.9) | 15.0  (9.9, 20.0) | 21.0  (15.5, 26.5) |
| Surf Coast† | 0.0  (0.0, 0.0) | 0.5  (-0.1, 1.0) | 2.9  (1.6, 4.2) | 10.1  (7.3, 12.9) | 29.6  (21.2, 31.9) | 13.1  (9.5, 16.8) | 6.9  (4.6, 9.2) | 7.7  (5.5, 10.0) | 5.1  (3.4, 6.9) |
| Warrnambool‡ | 1.4  (0.4, 2.3) | 0.8  (0.1, 1.5) | 4.7  (3.0, 6.5) | 21.8  (18.3, 25.4) | 22.6  (19.0, 26.2) | 21.7  (18.1, 25.2) | 21.2  (17.4, 24.9) | 18.2  (14.7, 21.7) | 15.2  (12.1, 18.3) |
|  | **45-49** | **50-54** | **55-59** | **60-64** | **65-69** | **70-74** | **75-79** | **80-84** | **85+** |
| Colac-Otway† | 12.5  (9.1, 15.9) | 9.6  (6.6, 12.6) | 5.6  (3.4, 7.7) | 7.4  (4.9, 9.8) | 2.4  (1.0, 3.8) | 4.2  (2.2, 6.3) | 2.7  (0.8, 4.6) | 6.4  (3.1, 9.8) | 7.6  (3.6, 11.5) |
| Corangamite† | 15.1  (10.9, 19.3) | 7.8  (4.8, 10.7) | 4.8  (2.6, 7.0) | 4.5  (2.3, 6.7) | 5.2  (2.7, 7.6) | 6.2  (3.4, 9.0) | 4.1  (1.5, 6.6) | 6.4  (2.6, 10.2) | 6.3  (2.4, 10.2) |
| Glenelg† | 12.8  (9.3, 16.3) | 9.7  (7.0, 12.5) | 7.9  (5.4, 10.4) | 6.0  (3.8, 8.2) | 6.0  (3.7, 8.3) | 7.6  (4.5, 10.7) | 3.8  (1.3, 6.2) | 3.8  (0.8, 6.9) | 5.4  (1.9, 9.0) |
| Greater Geelong‡ | 15.2  (14.1, 16.4) | 9.6  (8.7, 10.5) | 7.4  (6.6, 8.2) | 5.8  (5.0, 6.5) | 4.4  (3.7, 5.0) | 5.8  (5.0, 6.7) | 5.6  (4.6, 6.5) | 8.3  (6.9, 9.7) | 7.5  (6.4, 8.5) |
| Moyne† | 9.1  (5.9, 12.4) | 5.7  (3.2, 8.1) | 5.6  (3.2, 8.0) | 6.0  (3.5, 8.5) | 2.1  (0.6, 3.7) | 3.6  (1.1, 6.1) | 4.5  (1.2, 7.8) | 12.6  (5.8, 19.4) | 9.0  (3.4, 14.5) |
| Queenscliffe† | 35.3  (18.0, 52.6) | 5.4  (-0.7, 11.5) | 1.6  (-1.6, 4.8) | 0.0  (0.0, 0.0) | 0.0  (0.0, 0.0) | 1.1  (-1.1, 3.3) | 0.0  (0.0, 0.0) | 0.0  (0.0, 0.0) | 0.0  (0.0, 0.0) |
| Southern Grampians‡ | 19.9  (15.0, 24.8) | 7.1  (4.2, 9.9) | 7.3  (4.5, 10.1) | 4.6  (2.4, 6.8) | 2.4  (0.7, 4.0) | 5.2  (2.5, 7.9) | 7.2  (3.4, 11.0) | 5.5  (1.7, 9.3) | 6.4  (2.6, 10.1) |
| Surf Coast† | 5.7  (3.8, 7.5) | 2.1  (1.0, 3.3) | 3.4  (2.0, 4.9) | 0.5  (-0.1, 1.1) | 2.1  (0.9, 3.3) | 1.5  (0.3, 2.8) | 1.6  (0.0, 3.2) | 7.8  (3.6, 12.1) | 8.9  (4.4, 13.5) |
| Warrnambool‡ | 12.2  (9.6, 14.9) | 12.8  (10.1, 15.5) | 9.6  (7.2, 12.0) | 8.6  (6.3, 10.9) | 5.2  (3.3, 7.1) | 4.3  (2.4, 6.2) | 7.6  (4.6, 10.5) | 7.1  (3.9, 10.3) | 14.8  (10.4, 19.3) |

*Note: †=Data derived from Rural Acute Hospital Database Register; ‡=Data derived from Victorian Emergency Minimum Dataset.*
