# Supplementary material for: Mental health emergency presentations across the Barwon South West region in Victoria, Australia: An epidemiological investigation
Source: Emerg Med Australas. 2023 Apr 24;35(5):777–85. doi: 10.1111/1742-6723.14223 (PMC10947229; doi:10.1111/1742-6723.14223)
Supplement: Supplementary file 2 — Table S2. Types of mental health presentations according to local government area, based on ICD‐10‐AM principal diagnosis and intentional self‐harm. Data are presented as number (% [of total for local government area]) of types of mental health presentations. [file EMM-35-777-s002.docx]

**Supplemental Table 2.**

Types of mental health presentations according to local government area, based on ICD-10-AM principal diagnosis and intentional self-harm. Data are presented as number (% [of total for local government area]) of types of mental health presentations.

|  | **F00-F09** | **F10-F19** | **F20-F29** | **F30-F39** | **F40-F48** | **F50-F59** | **F60-F69** | **F70-F79** | **F80-F89** | **F90-F98** | **F99** | **Intentional**  **self-harm** |
| --- | --- | --- | --- | --- | --- | --- | --- | --- | --- | --- | --- | --- |
| Colac-Otway† | 25  (4.6) | 148  (27.0) | 66  (12.0) | 77  (14.1) | 167  (30.5) | 6  (1.1) | 9  (1.6) | _§_ | _§_ | 23  (4.2) | 26  (4.5) | _§_ |
| Corangamite† | 27  (6.6) | 81  (19.7) | 33  (8.0) | 48  (11.7) | 200  (48.7) | _§_ | _§_ | _§_ | _§_ | 5  (1.2) | 15  (3.7) | _§_ |
| Glenelg† | 14  (2.0) | 200  (29.0) | 84  (12.2) | 73  (10.6) | 259  (37.5) | _§_ | _§_ | _§_ | _§_ | 28  (4.1) | 28  (4.1) | _§_ |
| Greater Geelong‡ | 520  (7.0) | 2,350  (31.6) | 959  (12.9) | 1,097  (14.7) | 1,627  (21.9) | 60  (0.8) | 281  (3.8) | 24  (0.3) | 18  (0.2) | 203  (2.7) | 307  (4.1) | 55  (0.7) |
| Moyne† | 18  (5.3) | 80  (23.5) | 41  (12.0) | 42  (12.3) | 142  (41.6) | _§_ | _§_ | _§_ | _§_ | 9  (2.6) | 307  (4.1) | _§_ |
| Queenscliffe† | _§_ | 14  (58.3) | _§_ | 5  (20.8) | _§_ | _§_ | _§_ | _§_ | _§_ | _§_ | _§_ | _§_ |
| Southern Grampians‡ | 13  (2.7) | 145  (30.0) | 64  (13.2) | 38  (7.9) | 175  (36.2) | 12  (2.5) | _§_ | _§_ | _§_ | 14  (2.9) | 19  (3.9) | _§_ |
| Surf Coast† | 30  (6.6) | 160  (35.3) | 38  (8.4) | 94  (20.8) | 89  (19.7) | 6  (1.3) | 8  (1.8) | _§_ | _§_ | 15  (3.3) | 13  (2.9) | _§_ |
| Warrnambool‡ | 64  (5.3) | 309  (25.4) | 157  (12.9) | 128  (10.5) | 477  (39.2) | 15  (1.2) | 19  (1.6) | _§_ | _§_ | 28  (2.3) | 19  (1.6) | 10  (0.8) |
| Total*^¶^* | 712  (6.1) | 3,487  (30.0) | 1,443  (12.4) | 1,602  (13.8) | 3,139  (27.0) | 103  (0.9) | 325  (2.8) | 25  (0.2) | 19  (0.2) | 325  (2.8) | 433  (3.7) | 73  (0.6) |
| Chi-square (df),  *p*-value | 61.4 (8) *p*<0.001 | 143.4 (8)  *p*<0.001 | 85.4 (8)  *p*<0.001 | 56.6 (8)  *p*<0.001 | 388.3 (8)  *p*<0.001 | 22.1 (8)  *p*<0.05 | 61.7 (8)  *p*<0.001 | 0.3 (8)  *p*=1.00 | 0.2 (8)  *p*=1.00 | 19.7 (8)  *p*<0.05 | 36.4  *p*<0.001 | 7.4 (8)  *p*=0.49 |

*Note: F00-F09 Organic, including symptomatic, mental disorders; F10-F19 Mental & behavioural disorders due to psychoactive substance use; F20-F29 Schizophrenia, schizotypal, and delusional disorders; F30-F39 Mood (affective) disorders; F40-F48 Neurotic, stress-related, and somatoform disorders; F50-F59 Behavioural syndromes associated with psychological disturbances and physical factors; F60-F69 Disorders of adult personality and behaviour; F70-F79 Mental retardation; F80-F89 Disorders of psychological development; F90-F98 Behavioural and emotional disorders with onset usually occurring in childhood and adolescence; F99 Mental disorder not otherwise specified; †=Data derived from Rural Acute Hospital Database Register; ‡=Data derived from Victorian Emergency Minimum Dataset; §=data not reported due to <5 cases; ¶=Total includes all cases regardless of data not reported; df=degrees of freedom.*
